# Implementing Advance Care Planning for dialysis patients: HIGHway project

**DOI:** 10.1186/s12904-022-01011-5

**Published:** 2022-07-16

**Authors:** Giselle Rodriguez de Sosa, Amanda Nicklas, Mae Thamer, Elizabeth Anderson, Naveena Reddy, JoAnn Stevelos, Michael J. Germain, Mark L. Unruh, Dale E. Lupu

**Affiliations:** 1 Department of Medicine, University of New Mexico, Albuquerque, NM USA; 2 School of Nursing, George Washington University, Washington, DC, USA; 3 Medical Technology and Practice Patterns Institute, Bethesda, MD USA; 4 Pacific Institute for Research and Evaluation, Cullowhee, USA; 5 Supportive Kidney Care, Springfield, MA 01107 USA; 6 Renal and Transplant Associates of New England, PC, Springfield, MA 01107 USA; 7 Center of Aging, Health and Humanities, George Washington University, Washington DC, USA

**Keywords:** Advance Care Planning, Social workers, Dialysis patients, Implementation research, Patient- centered care, Shared decision making, Supportive care

## Abstract

**Background:**

Patients undergoing hemodialysis have a high mortality rate and yet underutilize palliative care and hospice resources. The Shared Decision Making-Renal Supportive Care (SDM-RSC) intervention focused on goals of care conversations between patients and family members with the nephrologist and social worker. The intervention targeted deficiencies in communication, estimating prognosis, and transition planning for seriously ill dialysis patients. The intervention showed capacity to increase substantially completion of advance care directives. The HIGHway Project, adapted from the previous SDM-RSC, scale up training social workers or nurses in dialysis center in advance care planning (ACP), and then support them for a subsequent 9-month action period, to engage in ACP conversations with patients at their dialysis center regarding their preferences for end-of-life care.

**Methods:**

We will train between 50–60 dialysis teams, led by social workers or nurses, to engage in ACP conversations with patients at their dialysis center regarding their preferences for end-of-life care. This implementation project uses the Knowledge to Action (KTA) Framework within the Consolidated Framework for Implementation Research (CFIR) to increase adoption and sustainability in the participating dialysis centers. This includes a curriculum about how to hold ACP conversation and coaching with monthly teleconferences through case discussion and mentoring. An application software will guide on the process and provide resources for holding ACP conversations. Our project will focus on implementation outcomes. Success will be determined by adoption and effective use of the ACP approach. Patient and provider outcomes will be measured by the number of ACP conversations held and documented; the quality and fidelity of ACP conversations to the HIGHway process as taught during education sessions; impact on knowledge and skills; content, relevance, and significance of ACP intervention for patients, and Supportive Kidney Care (SKC) App usage. Currently HIGHway is in the recruitment stage.

**Discussion:**

Effective changes to advance care planning processes in dialysis centers can lead to institutional policy and protocol changes, providing a model for patients receiving dialysis treatment in the US. The result will be a widespread improvement in advance care planning, thereby remedying one of the current barriers to patient-centered, goal-concordant care for dialysis patients.

**Trial registration:**

The George Washington University Protocol Record NCR213481, Honoring Individual Goals and Hopes: Implementing Advance Care Planning for Persons with Kidney Disease on Dialysis, is registered in ClinicalTrials.gov Identifier: NCT05324878 on April 11^th^, 2022.

## Background

Well documented deficiencies in the care of patients with kidney failure receiving dialysis treatment are associated with unnecessary suffering for these patients at the end of life (EOL). Patients with kidney failure experience high symptom burden, unmet psychosocial needs, lack of shared decision making and advance care planning, and very high rates of high intensity care at the end of life [1–3]. To address this gap, the prior research team developed the Shared Decision Making — Renal Supportive Care (SDM-RSC) intervention[4]. The SDM-RSC intervention was developed through qualitative interviews with advisory boards comprised of patients and stakeholders. The multi-modal intervention focused on goals of care conversations between patients and family members with the nephrologist and social worker [5–7]. It targeted deficiencies in communication, estimating prognosis, and transition planning for seriously ill dialysis patients.

The SDM-RSC was tested in a 1-arm longitudinal interventional cohort in 18 dialysis centers in Massachusetts and New Mexico. Among study participants, the advance directive completion rate and understanding of advance directives were substantially higher than in usual care; 75% of participants named and documented a healthcare proxy and 63% had physician orders for life sustaining treatment (POLST), in comparison to 49% with advance directives and 3% with POLST in usual care [8]. Among deceased study participants who engaged in an SDM-RSC meeting, 48% voluntarily withdrew from dialysis prior to death and 39% received hospice services (compared to the overall rate in these dialysis centers of 24.8%) [9]. The HIGHway project was developed to bring the original SDM-RSC intervention to scale, while updating it based on stakeholder recommendations, other recent research, and insights from the ongoing COVID-19 pandemic.

## Methods/design

Our implementation of SDM-RSC is designed to demonstrate scalability within organizations that serve 80% of all dialysis patients in the US. The major goal of the HIGHway project is to routinize ACP and make it an expected part of regular workflow in the care of patients undergoing dialysis. In collaboration with a newly formed stakeholder advisory board comprised of kidney patients, and health care providers the intervention has been renamed HIGHway to convey the purpose of the project during this implementation phase. The new project name embodies its goal: the way to “Honor Individuals Goals and Hopes”. HIGHway trains and supports dialysis center social workers or nurses to communicate with their patients about their hopes and goals for their future care plans. This process, known as advance care planning (ACP), helps relieve patient concerns about the future, lays the foundation for better goal concordant care at the end of life, and fosters a deeper connection between the patient and the dialysis care team.

The project objectives are to:

- Implement the HIGHway intervention with a project team consisting of a social worker or nurse at 50 clinics, and train them to conduct ACP using best practices.
- Assist social workers/nurses to implement ACP into their regular workflow with personal coaching, webinars, and multimedia teaching materials.
- Use a dedicated web-based application software to guide social workers/nurses on the ACP process of patients in dialysis centers and provide resources for holding ACP conversations.
- Provide ongoing coaching through monthly teleconferences to bolster social worker/nurse skills through case discussion and mentoring.
- Evaluate the ACP training received by the nurses and social workers.
- Develop a long-term implementation and scale-up plan for training social workers/nurses in ACP in different dialysis centers in conjunction with the Coalition for Supportive Care of Kidney Patients, Forum of End of Stage Renal Disease (ESRD) Networks, the National Council of Nephrology Social Workers, the National Renal Administrators Association, and dialysis organizations.

### Implementation strategy

In collaboration with three major dialysis organizations, we will implement the HIGHway intervention on a larger scale in terms of the number of sites, participants, and patients than in the original project. We also broaden the scope to include home dialysis patients, and to optionally be delivered via telehealth. Given the planned increase in home dialysis due to the American Kidney Health initiative, this adaptation to telehealth and to home dialysis patients will make our results more widely applicable to future expected composition of the dialysis population.

**Fig. 1:**
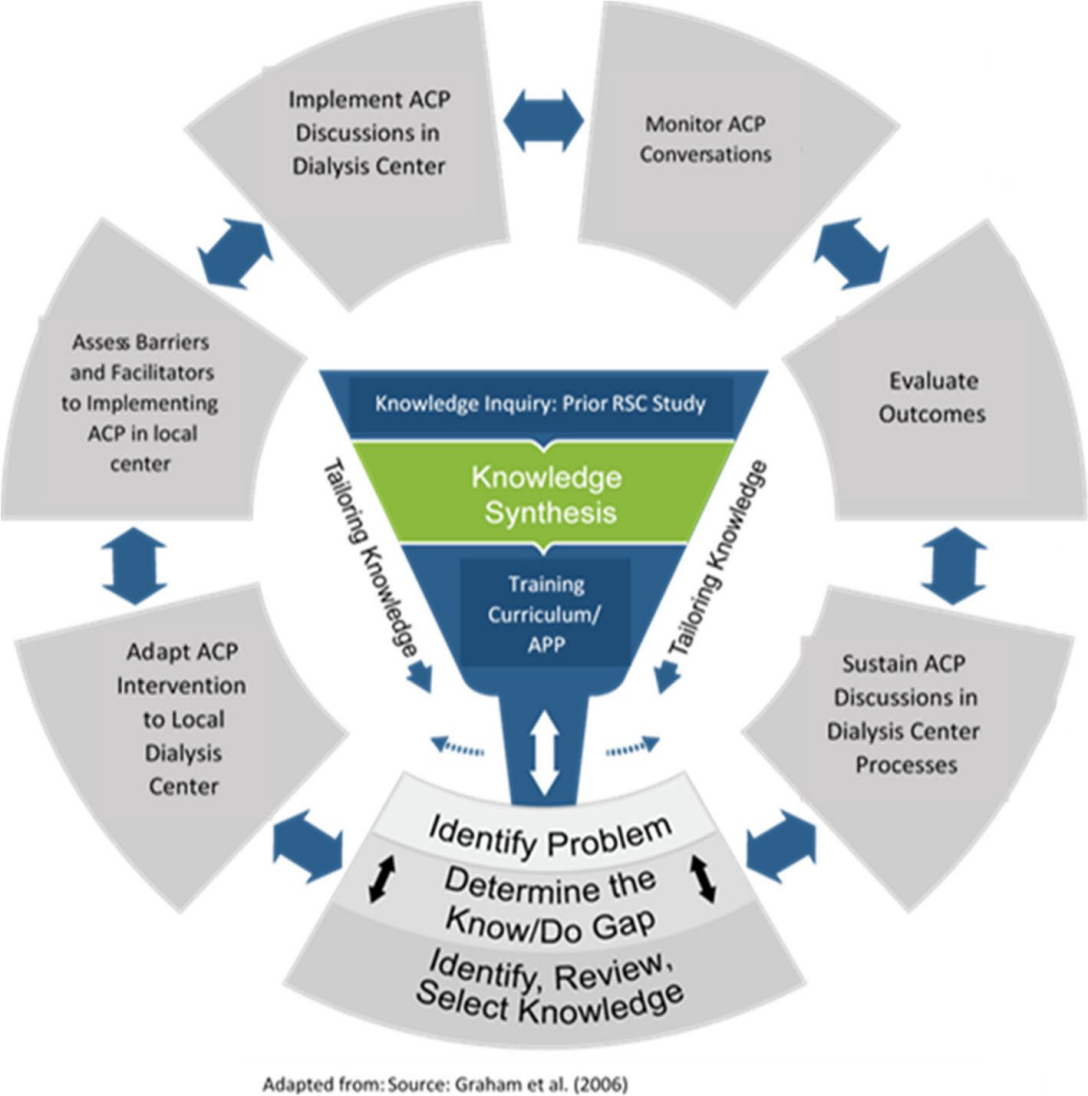
Knowledge to Action Framework [10]

**Fig. 2:**
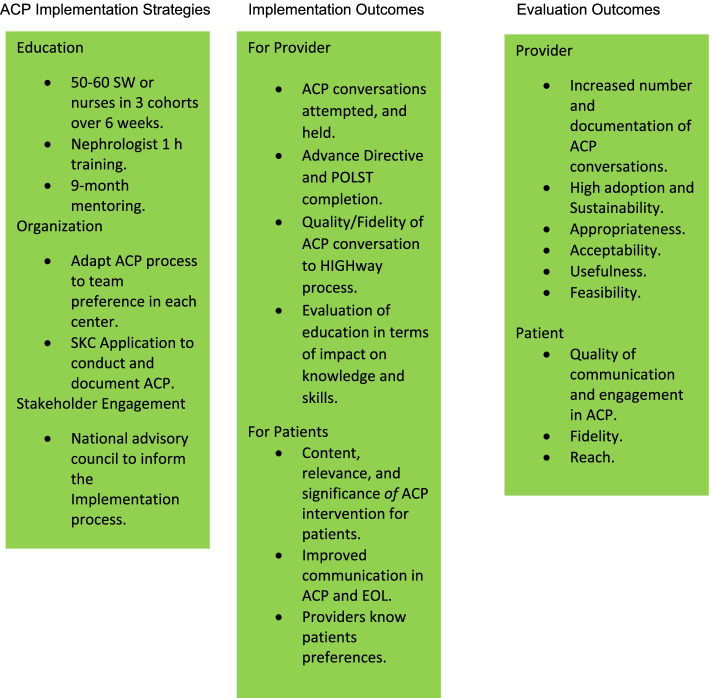
Logic Model for HIGHway Project Implementation

This study uses CFIR to guide both implementation and evaluation (Fig. 1. Knowledge to Action Framework [10]). The KTA framework cycle [11] is used within the intervention characteristic factor of CFIR to move the HIGHway intervention into the specific context of each dialysis center. The foundational logic model for achieving the goal-concordant care is adapted from the Sanders 2017 model for goal-concordant care [12] (Fig. 2).

HIGHway expands through both scale-up and scale-out on the previous SDM-RSC project intervention. Scaling up in numbers is achieved by enrolling a dialysis center team consisting of social worker or nurse, at 50–60 dialysis centers. Scaling-out happens by including a broader population of patients in the dialysis clinic instead of focusing solely on the seriously ill patients. The patient advisory council in the previous SDM-RSC study suggested of the inclusion of all patients within the dialysis center because they felt ACP would be of value to all dialysis center patients, they wanted ACP to be normalized as a regular part of care, and they wanted to avoid patients being alarmed by perceiving that they had been singled out for an “end of life” intervention.

Considering stakeholder advisory council recommendations and of the realities of implementation during the COVID pandemic, the social work training component of the SDM-RSC intervention has been streamlined and adapted to videoconference. Further, the computer application – a dedicated APP developed for this project – to guide documentation of the conversations has been made available on both Windows and IOS platforms. The overall aim is to increase the ability of the dialysis center teams to hold multi-disciplinary ACP conversations with all their in-center and home dialysis patients. A crucial component of this project is engagement of stakeholders throughout the process period. This will be accomplished through a national advisory council, chaired by the Coalition for Supportive Care of Kidney Patients. Key stakeholders, including patients, family members/caregivers, practitioners and organizational leaders will meet quarterly to inform the implementation process at each stage of the project.

### Intervention activities

The HIGHway intervention, adapted from the previous SDM-RSC, now contains the elements shown in Table 1. The core of the intervention is training social workers and/or nurses in the dialysis center in advance care planning, and then supporting them for a subsequent 9-month action period as they work to implement and refine the processes. Grounded in a Motivational Interviewing framework [13], the HIGHWay training adds to the standard practice of advance care planning discussion by introducing the elements of Stages of Change Theory [14], with a heavy emphasis on person centered approaches and empathy [15]. The training program’s pedagogical approach focuses on the didactic and experiential nature of learning, by offering skills-based training models for social workers and nurses. The training program is wholistic in its approach and extends beyond ACP to include modules on cultural sensitivity, spirituality, care coordination, and compassion fatigue and self-care.

**Table 1:** Key Implementation and evaluation activities of HIGHway project

Intervention	Activity
Implementation of each intervention	
Resources	• Provide a resource-rich app that allows teams to easily access advance care planning and kidney supportive care tools • Through app: guide workflow for advance care planning and follow up
Training	• Provide video conference training: ○ 6 h for social worker or nurse in role of advance care planning “coach” with patients (3 sessions of 2 h each over course of 6 weeks.) • Provide 6 h of social work CE for participation in training
Ongoing supervision	• Provide monthly mentoring/supervision group on-line for social workers and nurses. Use a case-based approach to build skills in advance care planning
Ongoing progress reports	• If site is using app for phone, iPad, or desktop, provide function on app to facilitate tracking and reporting completed conversations • In the alternative, social worker or nurse responds to short weekly email survey with 4 questions to capture ACP
Quality check of ACP discussions	• Faculty review recording and provide feedback to social worker/nurse on recorded ACP sessions • Provide constructive criticism and coaching to improve discussions
Evaluation after intervention	
Patient Mail-in Survey	• Provide IRB approved survey link and/or hard copy for mailing to be handed to patients who participate in ACP discussions
Social Worker/Nurse Online Survey	• Evaluation of training sessions includes both pre and post evaluation questions for comparison • Provide social worker survey (on-line) to evaluate participation in program, extent to which have adopted into ongoing workflow, identify facilitators and barriers, and assess future sustainability

Nephrologist and other clinic staff members will also have an opportunity to engage in both asynchronous and synchronous training. Training will focus on the HIGHWay model with opportunities to build skill around discussing ACP, and the role of the social worker, and importance of care coordination and interdisciplinary teamwork. Example video demonstrations and role plays will be used.

A computer application, The Supportive Kidney Care application (SKC App) enables dialysis center clinicians along the steps of an optimal ACP process. The first app was based on the Renal Physician Association “Shared Decision Making” Guidelines and provided tools to help dialysis center teams to assess prognosis and disease trajectory, guide discussion, and document goals of care with ACP forms. The revision of the app now follows the HIGHway roadmap and allows a more patient-centered, flexible process to occur and be documented. The app is optional for teams to adopt. We will evaluate the extent to which the app was useful and adopted by teams.

We work with the dialysis center teams to understand how ACP can be made a seamless part of regular workflow so that it does not require large amounts of additional resources. The process of folding ACP into existing workflow is part of the training and support provided to the dialysis center teams.

Around month 3–5 of the 9-month activity period, the social worker or nurse will select one patient to have an ACP conversation with and record the conversation with patient's consent. The lead social work faculty member will then hold an individual mentoring session with the social worker/nurse to provide supportive feedback to refine their communication skills.

### Recruitment and consent process

Three large dialysis organizations have agreed to collaborate to implement this initiative. We will have a champion role that may be filled by any nephrologist at the site interested and willing to be part of the project. We are working in collaboration with the administration of the collaborating dialysis organizations to recruit social workers or nurses to participate. The dialysis organizations set their own criteria for which centers will be eligible to participate based on organizational priorities (e.g., dialysis centers who have completed installation of EMR, dialysis centers in a certain region). The dialysis organization will contact their employees with information about the HIGHway project using standard internal communication strategies such as email lists. Alternatively, others who learn of the project via marketing efforts by the Coalition for Supportive Care of Kidney patients may individually choose to apply directly to the project and will be enrolled if space is available in the training cohort. Currently, HIGHway is in the recruitment stage.

Standard consent processes as approved by the IRB will be used to enroll in the project training, and patients who are approached to participate in an audio recorded interview. As approved by the IRB, the consent for the audio-recorded conversation involves obtaining verbal consent, with a waiver of documentation of consent for patients who agree to have an ACP conversation recorded. To obtain this consent, a member of the project team will set up a WebEx call with social worker/nurse and patient and will explain the project, answer questions, and obtain verbal consent to audio record the conversation and create a transcription from the audio record. Once both have consented to the recording, the project team member will begin the recording, but leave the WebEx session so that the patient will have privacy during their conversation. Processes for temporarily storing the recording, creating, and storing a transcript for analysis, have been approved by the IRB.

### Implementation timeline

This implementation study will enroll three cohorts of social workers and nurses beginning in early 2022. The cohorts will receive initial training over six weeks. Then each cohort will participate in a 9-month action period with ongoing coaching and mentoring in ACP conversations. Monthly calls to discuss additional topics along with implementation coaching will take place during the action period. Follow-up data collection will occur at the end of the action period and the project is scheduled to close May 31, 2023.

### Implementation outcomes

Our project will focus on implementation outcomes as well as selected patient and provider outcomes for intended project goals. Success will be determined by adoption and effective use of the ACP approach within in-center as well as home dialysis settings. Patient and provider outcomes will be measured by the number of ACP conversations held and documented; POLST completed; the quality and fidelity of ACP conversation to the HIGHway process as taught during education sessions; provider evaluation of education activities in terms of impact on knowledge and skills; content, relevance, and significance of ACP intervention for patients, and SKD App usage.

### Data collection

There are several distinct study populations for this study, with procedures and instruments for evaluation specific to each: 1) social workers/nurses participating in the training and implementation of intervention, 2) patients who agree to provide feedback on the advance care planning service they receive, 3) patients who agree to have an ACP session recorded.

Data on the number of ACP conversations conducted and the number of advance directives completed and filed in charts will be collected directly from the app for those teams that use it. In the alternative, sites that do not use the app will submit a brief weekly report via email with the number of ACP activities conducted that week as well as any successes or barriers encountered.

Fidelity check of the intervention will be performed by auditing a selection of the audiotapes of conversations between social works/nurses and patients to assess completeness of these conversations, to assess how adherent the visit was to the ACP checklist.

The anonymous survey for patients who attended an ACP session is based on previously validated Quality of Communication Scale, and adaptation of Patient Enablement Instrument. The survey’s purposes are to assess satisfaction with the ACP session, including change in confidence to complete ACP steps and quality of communication. Qualitative interviews of a sample of patients within dialysis center will be conducted to assess content, relevance and significance of ACP discussions based on a previously developed interview guide.

The implementation outcomes will be assessed through a survey to the participating social workers/nurses at the completion of their 9 months of participation. The questionnaire is based on the Workshop Evaluation Form (WEVAL). The questionnaire has been piloted with several social workers and adapted to the HIGHway project. Our main collection sources will be the evaluation questionnaires completed by the social workers or nurses, contingent on patients who consent to participate.

When an ACP conversation takes place, social worker/nurse will provide patient with an informational flyer about the project and a postcard with survey link. A Survey link will connect patient to a brief survey via Qualtrics (an encrypted survey system) regarding the conversation. Completion of survey will signify consent. The survey is anonymous.

We plan to enroll 60 social workers or nurses total over the several training cohorts. The turnover and dropout are expected to be about 17%, resulting in target of 50 social workers/nurses who complete entire 9 months of training. The rational is the training capacity of the project. We expect that approximately 1,600 patients will receive ACP service because we will target each social worker/nurse conducting one ACP conversation per week during the 9 months of training, beginning after the first month. The proportion of patients who will participate in the post implementation survey is unknown. The target is 25% participation rate.

Quantitative analysis will be primarily descriptive, with descriptive statistics with confidence intervals to characterize survey responses on various questions. For several of the knowledge gain questions asked before and after the social workers engage in training, chi-square and t-tests will be used to assess change. Qualitative analysis will be used for both inductively and deductively assessing themes and subthemes. Given the short 2-year time frame for this project, and the proposed provision of ACP services to both seriously ill as well as stable dialysis patients, we have not tried to measure the impact of these activities on our end-users in terms of healthcare utilization or specific health outcomes or choices. Instead, the success of our ACP intervention—and receipt of goal-concordant care as the gold standard— is inferred from the high-quality communication based on our intervention, as demonstrated by the fidelity check of audiotaped ACP conversations and by patient survey responses. Further, we will measure the success of this implementation project by documenting that all ACP-related activities and discussions have taken place, by assessing the quality and completeness of GOC discussions, by mail-in survey of patients and family participating in discussions, and by qualitative interviews with selected patients.

## Discussion

Patients on dialysis want to discuss their preferences for treatment at the end of life (EOL) [16], but few do so [16, 17] and most nephrologists are reluctant or feel unprepared to lead such discussions [18, 19]. Effective tools are critically needed to elicit such preferences since over 50,000 Americans die of kidney disease annually, more than from breast or prostate cancer [20]. For dialysis patients with significant co-morbidities, risk of death within a year of starting dialysis is stark. Of those who had 4 or more comorbidities, 26% died within 30 days of dialysis initiation, and 60% died within a year [21]. These patients have higher – and often unwanted – intensity of care at EOL; in a four-year study of the United States Renal Data System, 49% of elderly long-term hemodialysis (HD) patients spent time in an intensive care unit in their final month of life, compared with 24% of cancer patients [22]. Further, nephrology clinicians think they understand their patients’ priorities, but a majority in one study were wrong about whether their patient [23]. The SDM-RSC intervention was designed to systematically elicit patient preferences for EOL care so that preference-concordant care could be provided.

Meaningful EOL conversations can change these outcomes and are associated with increased hospice referral, less aggressive and expensive medical treatment, and higher levels of family satisfaction. Yet less than 10% of ESRD patients report having a conversation about any EOL issues with their nephrologist in the previous year, although 90% said such conversations were important [14]. Few patients with ESRD engage in ACP, and the vast majority lack a written advance directive or surrogate decision maker, leaving them unprepared to provide guidance in medical decisions in a crisis [19–23].

By virtue of their willingness to voluntarily participate in this project, the dialysis center teams, especially the social workers, are likely to be more open to advance care planning than the average dialysis center staff member. Early adopters likely have different motivations than later adopters. The results of this project will be generalizable to others with an early adopter mindset. To achieve further spread beyond early adopters, other encouragements (such as introducing regulatory requirements) may be needed. Various barriers were evident in the implementation of the original intervention. To address these barriers will require enhanced social worker/nurse and inter-professional training; increased readiness and comfort discussing prognosis amongst dialysis team members; early engagement at all participant levels; and routinization of the process through policy change—all issues we plan to address in this larger implementation project.

Unanticipated barriers that may arise will be addressed by the project team using creative problem solving as a team, including getting input from the stakeholder engagement group, the dialysis organization advisory group, and the participating center teams. The management strategy of outlining the pros and cons of options for responding to barriers will be used to decide on optimal solutions to unanticipated problems.

The implementation of SDM-RSC rapidly scales the multimodal intervention to improve advanced care planning in dialysis centers across the US. The goal of this project will be to routinize advance care planning and make it an expected part of regular workflow in the care of patients undergoing dialysis.

## Data Availability

All data will be storage in a secure manner. They include storing identifiable data only in secure RedCap database at GW or Qualtrics tool (https://www.qualtrics.com (https://www.qualtrics.com/)) at MTPPI, limiting access to the secure database to specified research staff on the IRB maintained study list, only providing de identified data to others outside the study team as needed for data analysis, destroying data after period of maintaining data has elapsed in accordance with GW policies. MTPPI network security starts from authenticating any user. Also, documentation of security policies, procedures and access records, information technology record of all configuration settings on the components of the network are kept and maintained. The data that support the findings of this study are available from MTPPI but restrictions apply to the availability of these data, which were used under license for the current study, and so are not publicly available. Data are however available from the authors upon reasonable request and with permission of MTPPI. For the Application software tool, the data is stored on Amazon Web Services (AWS) platform. According to AWS privacy statement document, it is a HIPAA compliant secure platform. We will further ensure data privacy by validating through a professional evaluation of the site and oversight of data storage and usage activities.

## Abbreviations

HIGHway: Honoring Individuals Goals and Hopes: Implementing Advance Care Planning for Persons With Kidney Disease on Dialysis
SDM-RSC: Shared Decision Making-Renal Supportive Care
ACP: Advance care planning
SKC: Supportive Kidney Care
EOL: End of life
POLST: Physician orders for life sustaining treatment
PCORI: The Patient-Centered Outcomes Research Institute
ESRD: End of Stage Renal Disease
IRB: Institutional Review Board
KTA: Knowledge to Action
CFIR: Consolidated Framework for Implementation Research
WEVAL: Workshop Evaluation Form
HD: Hemodialysis
